# The development of broad-spectrum antiviral medical countermeasures to treat viral hemorrhagic fevers caused by natural or weaponized virus infections

**DOI:** 10.1371/journal.pntd.0010220

**Published:** 2022-03-08

**Authors:** Mark R. Hickman, David L. Saunders, Catherine A. Bigger, Christopher D. Kane, Patrick L. Iversen

**Affiliations:** 1 Joint Project Manager for Chemical, Biological, Radiological, and Nuclear Medical (JPM CBRN Medical), Fort Detrick, Maryland, United States of America; 2 U.S. Army Medical Research Institute of Infectious Diseases (USAMRIID), Fort Detrick, Maryland, United States of America; 3 Logistics Management International Inc, Tysons Corner, Virginia, United States of America; 4 National Cancer Institute, Frederick, Maryland, United States of America; 5 Oregon State University, Corvallis, Oregon, United States of America; Center for Disease Control and Prevention, UNITED STATES

## Abstract

The Joint Program Executive Office for Chemical, Biological, Radiological, and Nuclear Defense (JPEO-CBRND) began development of a broad-spectrum antiviral countermeasure against deliberate use of high-consequence viral hemorrhagic fevers (VHFs) in 2016. The effort featured comprehensive preclinical research, including laboratory testing and rapid advancement of lead molecules into nonhuman primate (NHP) models of Ebola virus disease (EVD). Remdesivir (GS-5734, Veklury, Gilead Sciences) was the first small molecule therapeutic to successfully emerge from this effort. Remdesivir is an inhibitor of RNA-dependent RNA polymerase, a viral enzyme that is essential for viral replication. Its robust potency and broad-spectrum antiviral activity against certain RNA viruses including Ebola virus and Severe Acute Respiratory Syndrome Coronavirus 2 (SARS-CoV-2) led to its clinical evaluation in randomized, controlled trials (RCTs) in human patients during the 2018 EVD outbreak in the Democratic Republic of the Congo (DRC) and the ongoing Coronavirus Disease 2019 (COVID-19) pandemic today. Remdesivir was recently approved by the US Food and Drug Administration (FDA) for the treatment of COVID-19 requiring hospitalization. Substantial gaps remain in improving the outcomes of acute viral infections for patients afflicted with both EVD and COVID-19, including how to increase therapeutic breadth and strategies for the prevention and treatment of severe disease. Combination therapy that joins therapeutics with complimentary mechanisms of action appear promising, both preclinically and in RCTs. Importantly, significant programmatic challenges endure pertaining to a clear drug and biological product development pathway for therapeutics targeting biodefense and emerging pathogens when human efficacy studies are not ethical or feasible. For example, remdesivir’s clinical development was facilitated by outbreaks of Ebola and SARS-CoV-2; as such, the development pathway employed for remdesivir is likely to be the exception rather than the rule.

The current regulatory licensure pathway for therapeutics targeting rare, weaponizable VHF agents is likely to require use of FDA’s established Animal Rule (21 CFR 314.600–650 for drugs; 21 CFR 601.90–95 for biologics). The FDA may grant marketing approval based on adequate and well-controlled animal efficacy studies when the results of those studies establish that the drug is safe and likely to produce clinical benefit in humans. In practical terms, this is anticipated to include a series of rigorous, well-documented, animal challenge studies, to include aerosol challenge, combined with human safety data. While small clinical studies against naturally occurring, high-consequence pathogens are typically performed where possible, approval for the therapeutics currently under development against biodefense pathogens will likely require the Animal Rule pathway utilizing studies in NHPs. We review the development of remdesivir as illustrative of the effort that will be needed to field future therapeutics against highly lethal, infectious agents.

## Author summary

Development of medical countermeasures (MCMs) to treat and prevent viral infections with highly lethal filoviruses, such as Ebola virus (EBOV) and Marburg virus (MARV) is very difficult. Recently, US Food and Drug Administration (FDA)-approved vaccines and therapeutics for disease caused by EBOV and Severe Acute Respiratory Syndrome Coronavirus 2 (SARS-CoV-2) infections were obtained using the standard regulatory framework to address the new products’ safety and efficacy profile with randomized clinical trials. Clinical trials were feasible as sufficient numbers of patients were available due to the large scale outbreaks with both Ebola and SARS-CoV-2. However, the opportunity to conduct human studies against emerging viral pathogens is relatively rare, and effectively impossible for high-consequence pathogens likely to be used in biowarfare and bioterrorism, such as the filoviruses. The alternative regulatory pathway for development of new drugs targeting biodefense pathogens that cannot be conducted when human efficacy studies are not ethical and field trials are not feasible is through the FDA’s Animal Rule. In this review, we highlight how remdesivir’s preclinical development path, based upon the Animal Rule for an EVD indication, was leveraged to support its ultimate regulatory approval as an antiviral agent for the treatment of Coronavirus Disease 2019 (COVID-19). The preclinical and clinical development of remdesivir for both SARS-CoV-2 and filovirus treatment including evidence from Animal Rule and clinical studies is also reviewed. The challenges associated with employing the Animal Rule for development of multiple biodefense therapeutics against numerous and distinct viral biothreats within Biosafety Level 4 are discussed. The issues of developing pharmaceuticals for weaponized viruses are also examined, along with a proposed pathway for drug study and approval to meet FDA requirements.

## Introduction

Human filovirus infections are characterized by high morbidity and mortality in patients and exhibit case fatality rates ranging from 25% to 90%. The majority of infections have been associated with outbreaks of Ebola Zaire (ZEBOV), and minor outbreaks with other species of Ebola or the closely related Marburg virus (MARV) have also been noted [1]. While ZEBOV has been the most severe form observed clinically, the severity of the “lesser” filoviral hemorrhagic fevers (VHFs) in a deliberate aerosol attack may well exceed that of natural transmission. The probability for widespread panic and unrest underscore the potentially destabilizing influence of such an attack beyond its immediate medical impact [2,3]. While 2 new vaccines have been created for post- and preexposure prophylaxis to ZEBOV (VZV-ZEBOV, Merck; Ad26.ZEBOV/MVA-BN-Filo, Janssen Pharmaceuticals, Bavarian Nordic), no vaccine exists to prevent highly lethal infection by the other members of the EBOV family to include MARV. The most recent EBOV outbreak, which began in 2018 in the Democratic Republic of the Congo (DRC), led to 3,452 cases and 2,262 deaths, and the previous large outbreak in 2014 in Western Africa led to 28,600 cases and 11,325 deaths. Though typically smaller in size, past outbreaks of other filoviruses were also highly lethal; for example, MARV outbreaks have occurred with case fatality rates of up to 88% [1].

Filovirus infections are validated threats to the warfighter, and the development of medical countermeasures (MCMs) for prevention and treatment is a priority requirement [4]. The challenges of countering naturally occurring disease are compounded by those associated with deliberate use of VHF agents. Filoviruses from infected animals form stable aerosols that have been shown to infect other nonhuman primates (NHPs) in containment laboratory captivity [5,6]. While most remain theoretical in the absence of a confirmed deliberate attack, preliminary NHP studies of the natural history of aerosolized MARV and EBOV reveal devastating consequences. Multiple NHP models have demonstrated that aerosolized filovirus is not only uniformly infectious, but also comparably lethal to parenteral routes of infection [6–15]. Aerosolized Ebola has been shown to cause a rapidly progressive fatal disease within 4 to 5 days of exposure [1]. This is true not only for Ebola, but also for clinically milder VHF, such as Lassa fever [16].

We review the development of the antiviral remdesivir (Veklury) here to illustrate the challenges of fielding broad-spectrum biowarfare countermeasures, particularly those associated with development under the US Food and Drug Administration’s (FDA) Animal Rule [17]. Remdesivir is an antiviral drug invented and manufactured by Gilead Sciences, a US pharmaceutical company with expertise in antiviral drug development. Originally developed by Gilead as a candidate to treat viral hepatitis and respiratory syncytial virus infection, remdesivir was later studied for antiviral activity against multiple virus families, including filoviruses and coronaviruses [18]. The US military supported remdesivir development initially as a broad-spectrum antiviral countermeasure against Ebola infection. Remdesivir development interests also include treatment of MARV infections and aerosolized filovirus. While remdesivir did meet the Department of Defense’s (DOD) requirements for clinical efficacy, it did not outperform 2 monoclonal antibodies (mAbs) targeted specifically for Ebola in the four-arm comparator trial, which was conducted in the midst of a ZEBOV outbreak in the DRC in 2019 [19].

Remdesivir is active against a host of other viral threats, most notably Severe Acute Respiratory Syndrome Coronavirus 2 (SARS-CoV-2), where it recently obtained FDA licensure after demonstrated reduction in time to recovery in hospitalized Coronavirus Disease 2019 (COVID-19) patients [20]. While the Ebola epidemic and COVID-19 pandemic enabled clinical evaluation of remdesivir against 2 high-consequence viral pathogens in short order, the availability of patients infected with rare, emerging viral pathogens is expected to be the exception rather than the rule for therapeutics with biodefense-related indications. Biodefense countermeasures will need to continue to rely on the FDA’s Animal Rule for future development. The implications of this are discussed.

## Methods

The Joint Project Manager for Chemical, Biological, Radiological, and Nuclear Medical (JPM CBRN Medical), a subordinate element of the Joint Program Executive Office for Chemical, Biological, Radiological, and Nuclear Defense (JPEO-CBRND), funded preclinical research of a broad-spectrum antiviral therapeutic, remdesivir (GS-5734), with Gilead Sciences (Gilead). JPM CBRN Medical agreed to provide funding to support remdesivir preclinical development to assess its antiviral activity, which included laboratory tests to define its activity spectrum against biodefense viral pathogens of interest, including EBOV and MARV filoviruses. Later studies also included animal testing, conducted under Biosafety Level 4 conditions to evaluate remdesivir safety and efficacy and demonstrate positive proof-of-concept against filovirus infections. A significant portion of the preclinical testing was carried out at the US Army Medical Research Institute of Infectious Diseases (USAMRIID) in collaboration with Gilead. USAMRIID research demonstrated remdesivir’ s activity against Ebola in primates and generated data that could support further investigation of remdesivir as an Ebola treatment for humans [18]. The outcomes of these studies were originally intended to support development of the prototype product, remdesivir, initially for ZEBOV treatment via the FDA Animal Rule. This viewpoint article was based upon peer-reviewed manuscripts, US government (USG) reports and websites, scientific conference participation, and first-hand knowledge of the remdesivir development program by DOD personnel. No proprietary information is disclosed, nor is this review intended to convey the intent of the USG.

## Results

### a) Remdesivir pharmacology illustrates desired attributes for broad-spectrum antivirals

Remdesivir is an antiviral drug invented and manufactured by Gilead Sciences, a US pharmaceutical company with expertise in antiviral drug development. Remdesivir is a nucleotide prodrug inhibitor of viral RNA-dependent RNA polymerase (RdRp), a viral enzyme that is essential for viral replication. The chemical name for remdesivir is 2-ethylbutyl N-{(S)-[2-C-(4-aminopyrrolo[2,1-f][1,2,4]triazin-7-yl)-2,5-anhydro-d-altrononitril-6-O-yl]phenoxyphosphoryl}-L-alaninate [21]. Gilead’s preclinical research interest in addressing EBOV infections began as research collaborations with the Centers for Disease Control and Prevention (CDC) in 2013 and with the DOD’s USAMRIID in 2014 [18]. Gilead scientists selected compounds for testing against emerging viruses based on their proprietary knowledge about the compounds’ antiviral profiles. CDC and USAMRIID scientists investigated collections of Gilead’s small molecule inhibitors in a blinded fashion for antiviral activity against Ebola and other pathogens. These collaborations took place at the time of several outbreaks of Ebola virus disease (EVD) affecting multiple countries during the beginning of the 2014 West Africa EBOV outbreak. The CDC and USAMRIID were important collaborators as most of the research took place at these agencies’ Biosafety Level 4 facilities. The USAMRIID’s collaboration with Gilead involved the screening of more than 1,000 Gilead-owned compounds, tested in live cells to NHP evaluation with live, authentic ZEBOV for antiviral activity against multiple viruses. As the research progressed, the USAMRIID collaborated closely with Gilead scientists to advance multiple promising compounds, including remdesivir, from in vitro testing to nonclinical animal testing in NHPs.

Remdesivir is an adenosine analog nucleotide prodrug designed with a phosphoramidate (P-N) modification that facilitates passive prodrug diffusion into target cells. Delivery of the monophosphate nucleoside moiety thereby bypasses the initial rate-limiting phosphorylation step in nucleotide metabolism. Remdesivir distributes into cells where it is metabolized to the nucleoside monophosphate intermediate catalyzed by carboxyesterase 1 and/or cathepsin A, depending upon the cell type, and a phosphoramidase. Nucleoside analogs require further phosphorylation by intracellular nucleotide and nucleoside diphosphate kinases to create the triphosphate active form of the drug. Remdesivir triphosphate acts as an analog of adenosine triphosphate (ATP) and competes against the endogenous natural ATP pool with high selectivity (3.65-fold) over the natural ATP substrate for incorporation into nascent RNA chains [21]. The prodrug strategy produces an antiviral inhibitor with radically increased potency from the micromolar (for GS-441524, nucleoside) to the nanomolar range (for remdesivir triphosphate GS-443902) against several filoviruses [24–27]. Subsequent in vitro testing of GS-5734 (remdesivir) revealed activity in the nanomolar range against other filoviruses (other species of Ebola such as Tai Forest, Bundibugyo, Sudan) and MARV [21]. In addition, potent activity was found against other virus families including paramyxoviruses such as measles virus (MV), parainfluenza type 3 virus (PIV3), Nipah virus (NiV), Hendra virus (HeV), and against respiratory syncytial virus (RSV) [28–30] (see Table 1).

**Table 1:** Potency of remdesivir against a variety of viral targets.

Virus	EC50 (uM)
ZEBOV (Makona) [24] (primary macrophages)	.086
ZEBOV (Mayinga-GFP reporter virus) [30]	.066
MARV (bat 371 reporter virus) [30] (Huh7 cells)	.019
SARS-CoV-2 [21] (human airway epithelial cells)	.010

MARV, Marburg virus; SARS-CoV-2, Severe Acute Respiratory Syndrome Coronavirus 2; ZEBOV, Ebola Zaire.

Nucleoside/nucleotide analogs (NAs) have different mechanisms of action on viral DNA-dependent and RNA-dependent RNA polymerases by inducing chain termination, directly inhibiting the viral polymerase, or by acting as mutagens in the viral genome [25]. There are 2 mechanisms of action for the active metabolite of remdesivir (the triphosphate form of GS-443902); the first mechanism is a delayed chain termination at either position i + 5 for respiratory syncytial and EBOVs RdRps [28] or i + 3 for the highly pathogenic coronaviruses (SARS-CoV, SARS-CoV-2, and MERS-CoV) [28,31]. A second mechanism of action has been shown for remdesivir involving template inhibition of the SARS-CoV-2 RdRP [32]. Delayed or “leaky” chain termination was also reported for NiV (paramyxovirus family) [33]. Incorporation is highly efficient and nearly equal to its ATP cellular counterpart [28,31]. To date, both in vitro and in vivo studies with remdesivir have not reported development of antiviral resistance with the exception of 2 amino acid substitutions (F476L and V553L) in a conserved finger domain of the nsp12 RdRp gene of murine hepatitis virus (MHV), another coronavirus [34]. Engineered amino acid substitutions at conserved sites in the SARS-CoV nsp12 conferred resistance in vitro, but as with MHV, the variants resulted in an attenuated virus in vivo [34].

### b) Preclinical characterization of remdesivir

Exploratory animal studies were initiated with remdesivir at the USAMRIID to determine an effective dose against EBOV infection and to establish the treatment window against EBOV using rhesus macaques. A recent natural history study of EVD in rhesus monkeys demonstrates how intramuscular (IM) exposure to 1,000 plaque-forming units (PFUs) of EBOV manifests consistently across infected animals and culminates with mortality events occurring 7 to 10 days after exposure [35]. Therapeutic efficacy studies were conducted in which rhesus macaques were experimentally infected by parenteral administration of EBOV and subsequently treated with remdesivir using a loading dose of 10 mg/kg followed by once-daily treatment at 3 mg/kg for up to 12 days [24]. Treatment on day 3 postinfection was associated with 100% survival of Ebola-infected animals treated with remdesivir versus none of the Ebola-infected, untreated animals [24]. Additional studies have refined and broadened the treatment window by extending the time of remdesivir administration to longer intervals after infection using different filovirus and NHP species. A recent report describes administration of remdesivir beginning 4 or 5 days after MARV infection in cynomolgus macaques. Remdesivir administered once daily with a 10-mg/kg loading dose followed by a 5-mg/kg maintenance dose for 12 days provided an 83% survival outcome [36]. An additional study reported by a different research group found that remdesivir administration using the same 10/5 mg/kg for 12 days initiated 5 days postinfection provided 80% survival using a MARV challenge model using rhesus macaques [37].

Transmission of EBOV by small-particle aerosols has been documented in experimental settings using NHPs [38]. Rhesus monkeys exposed to aerosol doses of up to 1,590 PFUs of EBOV die 7 to 10 days postexposure. Viral RNA is detected in the lungs and tracheobronchial lymph nodes by day 3 postexposure, and serum viral RNA levels of >10^6^ PFU equivalents are detected on days 4 to 6 postexposure. The characteristics of EVD in aerosol exposure (AE) studies in NHPs are nearly identical to those in IM exposure studies with regard to survival, time to death, viremia peak, and infectious virus profile [39]. A rhesus macaque EBOV challenge study comparing remdesivir to vehicle alone was recently conducted using aerosol challenge. In this study, remdesivir treatment initiated 4 days after aerosolized EBOV exposure was associated with survival benefit, significant reduction in serum viral titer, and improvements in clinical pathology biomarker levels and lung histology compared to vehicle treatment (see Table 2). Importantly, these results showed similar outcomes to challenge studies conducted with IM inoculation [39]. Significantly greater EBOV replication was found in the lungs in the inhalational route compared with prior published studies using the IM route at the same lab [35]. This feature is of particular importance when considering MCM development against weaponized filoviruses delivered by aerosol.

**Table 2:** Efficacy of remdesivir in NHPs challenged with ZEBOV and MARV.

Animal Model	Route of Administration	Challenge Virus	Challenge Day	Outcome
Rhesus macaques [24]	IM	1,000 PFU Ebola Kikwit strain	Day 3 postinfection	100% efficacy
Rhesus macaques [39]	Aerosol	100 PFU Ebola Kikwit strain	Day 4 postinfection	67% efficacy
Cynomolgus macaques [36]	IM	1,000 PFU MARV Angola strain	Day 4 postinfection	83% efficacy
Rhesus macaques [37]	IM	1,000 PFU MARV Angola strain	Day 4 postinfection	80% efficacy

IM, intramuscular; MARV, Marburg virus; NHP, nonhuman primate; PFU, plaque-forming unit; ZEBOV, Ebola Zaire.

The findings in the aerosolized Ebola model raise important questions regarding the optimal route of administration based on desired sites of therapeutic effect in the body. Vigorous humoral and cellular responses in lung tissue in particular are likely to be a critical first line of defense. Intranasal IgA mAbs may have advantages over conventional IgG monoclonals administered intravenously. The same may hold true for vaccines. In the case of thereutics, strategies to target small molecules to the lung should be considered. In a similar vein, the study raises important questions regarding the comparability of parenteral routes of inoculation to aerosolization of agents in animal models. While Ebola behaved similarly between IM and inhalational routes in the NHP model, other biowarfare agents cannot be expected a priori to do the same.

### c) Human safety studies

The safety and pharmacokinetics of remdesivir were studied in 131 subjects in 4 healthy volunteer studies [21]. In general, remdesivir has been found to be safe and well tolerated. Half-life is approximately 1 hour following IV dosing, with maximum concentration reached at 40 minutes with roughly 90% of the drug protein bound. It is metabolized by CES1, Cathepsin A, and cytochrome P450 3A enzymes. While formal interaction studies have yet to be reported, an antiretroviral interaction study (NCT04385719) is currently underway in Uganda [40]. Adverse reactions occurred in 13% to 19% of healthy volunteers leading to treatment discontinuation in 2%, with both 5- and 10-day courses of therapy. Mild increases in alanine aminotransferase (ALT) occurred in nearly half of healthy volunteers in one small Phase I trial, but resolved after therapy was completed. Findings in humans differed to some degree from preclinical safety studies, which found evidence of renal injury in rat and NHP models. Remdesivir is not recommended in patients with a glomerular filtration rate below 30 mg/dL based on serum creatinine levels. Testing for both renal and hepatic dysfunction is recommended, the latter based largely on human safety studies. Subsequent safety studies in man have shown that the risk of administering remdesivir to patients with acute kidney disease is far less than originally thought [41,42]. Filoviral studies have included randomized active comparator trials for treatment of acute ZEBOV (Ituri strain) disease in the DRC. In addition, there have been various World Health Organization (WHO)-sponsored monitored emergency use of unregistered interventions (MEURIs) compassionate use protocols for treatment of Ebola-infected patients [19]. At the time of this publication, thousands have been dosed with remdesivir for EBOV and SARS-CoV-2, and remdesivir has been approved for the latter indication [21]. The dose studied for treatment of acute Ebola infection is a loading dose of 200 mg IV on day 1 with maintenance treatment at a dose of 100 mg IV for up to 14 days [19]. There were no significant serious adverse events (SAEs) observed in a small placebo-controlled study in 38 healthy male EVD survivors in Liberia and Guinea given a 5-day course of 100 mg IV remdesivir daily to eradicate the carrier state [43].

Remdesivir has been administered to tens of thousands of patients as treatment for COVID-19, at hundreds of sites in regulated clinical trials or compassionate use administration. Diarrhea, hepatic and renal abnormalities, rash, and hypotension, the latter as a precursor to SAEs including septic shock and multiorgan failure, have been the most common reported outcomes. In uncontrolled compassionate use settings, 23% had SAEs, many likely related to the underlying disease, with 8% discontinuing therapy due to side effects [44]. Adverse events for the most part do not seem to have been elevated over control groups treated with various standards of care (SOCs). Relatedness to remdesivir, however, has proven to be challenging to ascertain with greater than 60% of patients in both arms reporting adverse events in at least one study [45]. However, a larger percentage taking remdesivir suffered treatment-limiting adverse events compared to controls.

### d) Efficacy against Ebola virus—The PALM study

The Pamoja Tulinde Maisha (“Together Saving Lives”, PALM) study was a randomized controlled trial (RCT) in the DRC comparing 4 EBOV therapeutics to include the ZMapp mAb cocktail, the mAb mAb114 (Ebanga), the Regeneron mAb cocktail REGN-EB3 (Inmazeb), and remdesivir [19]. The results of the PALM study in the early diagnosis cohort (cycle threshold over 22) showed efficacy of 89% for mAb114, 91% for REGN-EB3, 69% for remdesivir, and 75% for ZMapp. The results for patients in the late diagnosis cohort (cycle threshold under 22) were as follows: 31% for mAB114, 36% for REGN-EB3, 14% for remdesivir, and 15% for ZMapp [19]. The overall survival rates were as follows: 65% for mAb114, 66% for REGN-EB3, 47% for remdesivir, and 50% for ZMapp; the overall survival rate for patients who received supportive care was 32%.

The 2 antibody therapeutics, REGN-EB3 and mAb114, were found to provide a statistically significant mortality benefit against acute EVD compared to ZMapp and remdesivir. The difference in the results for the early and late diagnosis cohorts supports the argument that treatment with any antiviral therapy for filovirus disease must start early to have the highest probability of success. While this remains to be proven clinically, poor outcomes in late presenting patients in PALM support the limitations of delayed antiviral monotherapy later in the disease course as immunologic dysfunction begins to predominate [46]. Combination therapy of an antibody-based therapy combined with an antiviral therapy may improve efficacy for both early and late stage patients as well as possibly prevent persistent infection.

Remdesivir inhibits viral replication by the virus’ RdRp. Remdesivir likely reduces viral load but not before or within enough time to prevent clinical decline in treated individuals in the PALM study. The study outcomes in the PALM trial raise several questions regarding direct-acting antiviral (DAA) treatment, including the need to understand the relationship between the required proportional drop in infectious virus to realize a sustained response with clinical improvement. Madelain and colleagues modeled viral kinetics in NHPs during therapy with DAAs and indicated that current DAAs do not achieve the viral load reduction required at day 5 (mimicking severe disease in humans) to provide 100% survival [23]. Madelain and colleagues stated that, in order to achieve effectiveness, a DAA needs to be administered at least 2 days prior to peak viremia and cytokine storm in NHPs and extrapolated to human infections. Ideally, obtaining viral kinetic data from patients with intensive sampling would be vitally important in understanding clinical benefit of remdesivir and the window of therapeutic efficacy.

The difference in the results for the early and late diagnosis cohorts supports the argument that early treatment with any antiviral therapy for filovirus disease must start early to have the highest probability of success. Combining an antibody-based therapy with an antiviral is likely to be additive for improving efficacy for both early and late stage disease. Only remdesivir has broad-spectrum activity against other filoviruses in animal models. Additionally, the ability to enter key immune sanctuary sites such as the brain and testes may have benefit against disease sequelae in survivors. A small placebo-controlled study of EBOV eradication from the semen among 38 survivors in Liberia and Guinea was recently published. Remdesivir was shown to significantly reduce persistent EBOV infection in the Partnership for Research on Ebola Virus in Liberia (PREVAIL) IV study indicating bioavailability in the testes [43]. A recent publication in the *New England Journal of Medicine* documented a transmission chain from a survivor of Ebola infection treated with mAb114. The patient suffered a recrudescent infection 149 days after initial discharge, transmitted his infection to his spouse (likely through sexual contact), and ultimately, this chain of transmission led to the infections of 91 cases in 6 health zones over a period of 4 months [47]. Treatment with a directly acting antiviral drug such as remdesivir during this patient’s hospitalization might have prevented the recrudescence of disease and subsequent transmission to others.

Furthermore, combination or sequential therapy may prevent post-EVD sequelae and development of poorly understood persistent infections by eradicating EBOV localized in immunologically privileged sites. A combination therapy study in patients has not been conducted to date, and the Ebola outbreak has since subsided, suggesting that an NHP bridging study might be useful. Interestingly, Cross and colleagues recently reported that combination therapy consisting of remdesivir and a human antibody targeting MARV achieved 80% protection against advanced disease in a MARV challenge model using rhesus macaques while remdesivir only or mAb only treatment yielded no survivors [37]. While two of the antibody therapies in the PALM trial showed the greatest clinical benefit for treatment of ZEBOV infections, both are specific for ZEBOV with no activity against other filoviruses.

Other considerations for use of remdesivir as a filovirus DAA include the following: (1) detection and treatment for the warfighter likely will be initiated earlier and result in greater efficacy; (2) EBOV mAb treatments still have an overall mortality rate of 30% at the affected population level (which may be decreased for warfighters promptly diagnosed and treated); (3) EBOV mAb monotherapy likely will not prevent all sequelae, including persistence; (4) remdesivir treatment may benefit EBOV survivors with persistent virus replication; and (5) combination or sequential therapy may be additive and improve overall outcomes including preventing persistent infection.

### e) Preclinical and clinical studies of remdesivir against SARS-CoV-2

Specific studies against coronavirus infections in vitro demonstrated activity against coronaviruses isolated from bats and circulating human coronaviruses in primary lung cells. In vivo rodent studies of SARS-CoV-2 pathogenesis have shown prophylactic and early therapeutic administration of remdesivir significantly reduced lung viral load, improved clinical signs, and enhanced respiratory function [48]. Subsequent in vitro studies have shown that remdesivir is active against a broad array of contemporary human coronaviruses and also against porcine deltacoronaviruses [49]. In rhesus monkeys, once-daily IV administration of remdesivir initiated 1 day prior to Middle Eastern Respiratory Syndrome Coronavirus (MERS-CoV) infection reduced lung viral load, improved clinical disease signs, and reduced lung pathology [50]. A recent publication demonstrated the efficacy of remdesivir for both prophylactic and treatment indications against MERS-CoV in rhesus macaques [51]. Prophylactic treatment initiated 24 hours prior to inoculation prevented MERS-CoV disease, and treatment with remdesivir 12 hours postinoculation provided a clear clinical benefit with reduction in symptoms, decreased viral replication, and decreased lung lesions [51]. A publication in *Nature Cell Research* has shown that both remdesivir and chloroquine effectively inhibit the SARS-CoV-2 virus in vitro (subsequent clinical studies did not show efficacy against COVID-19 in man) [45]. Taking all of these data together, remdesivir has shown in vitro and in vivo efficacy against a wide range of coronaviruses, including rapidly emerging zoonotic viruses (SARS and MERS-CoVs), which have shown pandemic potential.

A number of clinical studies of remdesivir against COVID-19 have been conducted at various sites around the world using remdesivir alone or in combination with other drugs. One of the largest studies completed thus far is the Adaptive COVID-19 Treatment Trial (ACTT-1) study sponsored by the National Institutes for Allergy and Infectious Disease (NIAID). ACTT-1 compared remdesivir treatment to placebo in hospitalized COVID-19 patients with lower respiratory tract involvement. There were 1,063 patients randomized (541 remdesivir versus 522 placebo), with a median recovery time of 11 days for remdesivir versus 15 days with placebo. Kaplan–Meier estimates of mortality by 14 days were 7.1% with remdesivir and 11.9% with placebo [20]. Gilead conducted 2 additional trials (the SIMPLE studies), which showed similar clinical benefit associated with 5 days of remdesivir treatment and 10 days of remdesivir treatment in severe COVID-19 patients [52,53]. The data from these 3 studies supported FDA approval for COVID-19 treatment in the US [54]. One additional study conducted by WHO, Solidarity, did not demonstrate similar efficacy for remdesivir in hospitalized patients. While randomized, the Solidarity study covered a large number of sites with significant variability in SOCs. The basis for observed differences in outcomes between the 2 trials is not clear; one possibility is the critical factor of time to initiate treatment after hospitalization.

### f) Combination therapy

Antiviral drugs require early treatment initiation for maximum efficacy [48]. DAA treatment earlier during the infection or prior to peak viral loads likely improves efficacy and patient outcomes. The possibility that there is substantial available replication space (uninfected cells), even at the time of death in COVID-19 patients, suggests that pathophysiology results from more than simply viral replication [55,56]. Antivirals may be less effective at the pulmonary stage of infection—typically between 5 to 10 days after onset of symptoms, but more effective earlier in the course of disease. Optimal treatment at later stages will likely require additional host-directed therapies. Gaps in understanding of COVID disease etiology include understanding the relative contributions of viral load, target cell depletion, and immunologic response affect pulmonary function degradation over time. The relative effectiveness of antivirals at the pulmonary stage of infection remains of questionable value given that pathophysiology has transitioned from viral replication to immunologic dysfunction. Delayed presentation of disease in clinical trials of Ebola has supported the notion that antiviral treatment must be initiated as early as possible before dysfunctional immune responses predominate [19,22].

Combined therapy of remdesivir with Janus kinase (JAK) inhibitor baricitinib (ACTIV) recently reported a reduction in average hospital stay from 8 to 7 days for the combination, and 18 to 10 days for those on high-flow oxygen or receiving noninvasive ventilation [57]. Remdesivir has been studied in combination with the IL-6 inhibitor tocilizumab (Remdacta); however, the primary endpoint was not met [58]. Toculizumab has been found to be of significant benefit over placebo when used alone in a randomized study, though not in a retrospective study [59,60].

### g) mAb therapy not recommended for hospitalized patients with SARS-CoV-2 infection

The results of the ACTIV-3 trial investigating treatment of COVID patients with mAbs have found that SARS-CoV-2 mAb products in development may be harmful in more advanced disease and use is not recommended in hospitalized patients by the NIH or Infectious Disease Society of America [61–63]. Bamlanivimab, in combination with etesivimab, was granted an Emergency Use Authorization (EUA) for treatment of mild and moderate COVID-19 disease only because of poorer outcomes seen in patients with more severe disease [64,65]. This highlights potential limitations of antibody-based therapies, particularly in a deliberate aerosol attack, and underscores the need for ongoing complementary broad-spectrum antiviral development [22]. Direct-acting agents may also have limitations in timing of administration, and questions of benefit as time from disease onset increases are critically important for high-consequence pathogens.

### h) Antibody-dependent enhancement during filovirus infection

Antibody-dependent enhancement (ADE) of filovirus infection has been demonstrated in mAbs derived from human survivors of Ebola infection [66]. All of the filovirus Ab therapeutics to include neutralizing mAbs and convalescent plasma target the filovirus glycoprotein (gP). It has unique properties among virus gPs in that it has multiple functions for the virus life cycle in the host. EBOV gP is heavily and unusually glycosylated with half of the molecular weight of the monomer attributed to N- and O-linked carbohydrates [67]. Furthermore, EBOV gP is C-mannosylated, and a truncated variant of gP, shown to cause immunopathology, is secreted during infection [68–70]. EBOV gP may even activate complement via the lectin pathway demonstrating that ADE (with convalescent plasma and mAbs) targeting multiple filoviruses is possible and independent of Ab subclass, neutralizing capability, or even epitope specificity [66].

In contrast to these published reports, Bournazos and colleagues suggested that the observed ADE published by others is an in vitro phenomenon not replicated in vivo [71]. The route of challenge and organ-specific responses may play a significant role in the development of immunopathology. Whether this occurs in humans is difficult to study, and, indeed, there are few recorded autopsies on filovirus-infected patients. This potentially serious, adverse event with therapeutic mAbs should be examined in detail to determine safety once Ab concentrations fall below neutralizing levels. Likewise, the potential for immunopathology extends to vaccines and also should be examined in detail using multiple routes of exposure given the biowarfare threat component of filoviruses. ADE with Ebola has not been well documented yet and remains a theoretical possibility at this time. It is likely to depend on factors including persistence of administered mAbs, the patients own immune response combining to provide an effective neutralization response.

The PALM trial underscored the need for very early recognition and initiation of specific therapy as mortality increased with time following onset of therapy [19]. In addition to rapid onset of fatal disease, the possibility for ADE with severe lung pathology must be considered. While there was no overt evidence of clinical ADE of disease in PALM based on results published to date, this principal has been demonstrated ex vivo in samples obtained from survivors of ZEBOV, MARV, and Bundibugyo virus [66,72].

### i) Comparison of human and nonhuman pathophysiology resulting from EBOV and MARV infection

**Fig 1:**
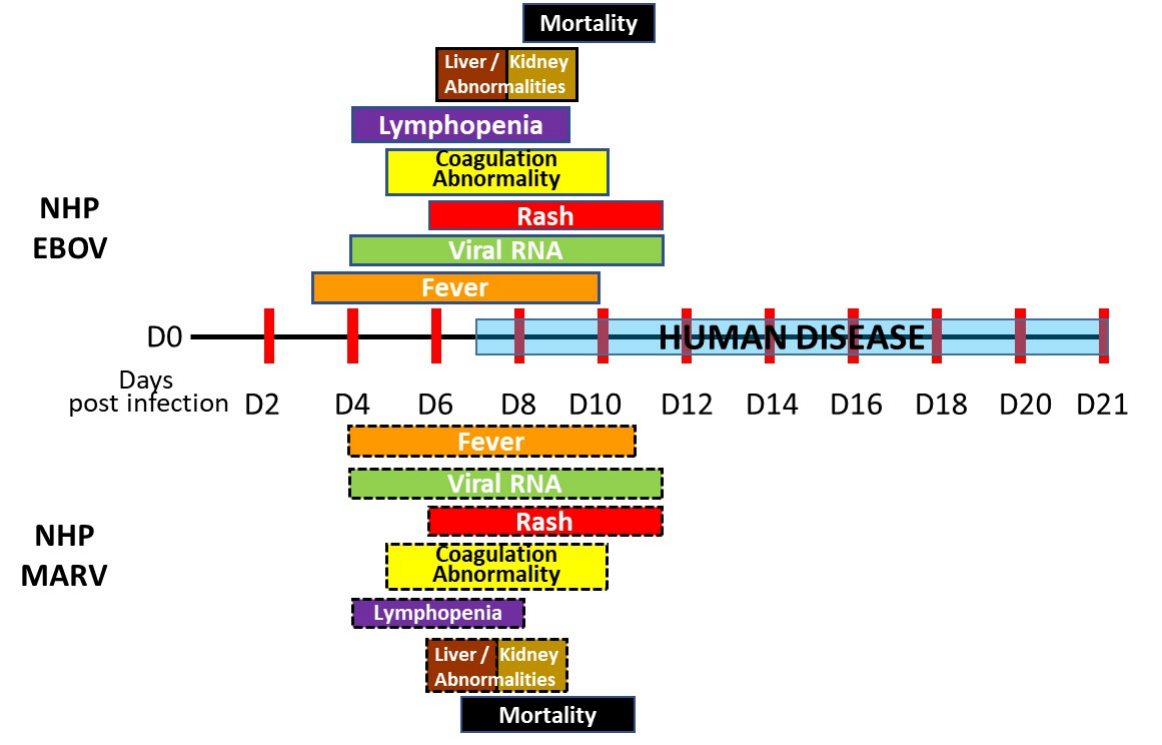
Comparison of EBOV and MARV pathophysiology over time. EBOV, Ebola virus; MARV, Marburg virus; NHP, nonhuman primate.

EBOV- and MARV-induced diseases, in both humans and NHPs, are similar in presentation, pathology, and mortality (Fig 1) adapted from Shifflett and colleagues [73]. However, disease course in NHPs is accelerated relative to the human clinical course. Furthermore, the course of disease, and primary and secondary endpoints following EBOV and MARV infection of NHP, overlap regardless of challenge route, challenge dose, and NHP model. Disease onset is rapid, and 83% to 100% of the control animals die within 6 to 8 days postchallenge. The majority of data have been obtained from studies involving the IM route of challenge, which, in turn, has provided the most mature filovirus NHP models to date and reproducibly induces critical, uniform endpoints of disease in both EBOV and MARV studies [10,11,14,35,64,73–79].

- IM inoculation produces consistent infection in NHPs.
- IM injection allows for delivery of a consistent quantity of virus among animals and between studies.
- IM inoculation produces key disease manifestations that closely resemble those occurring in fatal human cases.
- The FDA has indicated a preference for IM studies in the critical pathway for licensure of filovirus MCMs because of the above considerations.
- To date, no Good Laboratory Practice (GLP) studies utilizing an inhalational aerosol challenge have been conducted in a Biosafety Level 4 setting.

Consequently, the IM model may be an acceptable route of exposure for determination of filovirus MCM efficacy supporting the Animal Rule licensure pathway by the FDA. It is important to note that this may not be the case with other biothreat agents and aerosol challenge represent a more physiologically relevant challenge route for biodefense-related product development.

Although not required for expensive, complex, and regulated pivotal studies under the Animal Rule, we argue here that products being developed for a biodefense indication should include one or more aerosol challenge studies as part of the development package. An aerosol study will help elucidate lung or potentially other respiratory-related differences in pathogenesis. Studies should be conducted preferably in NHPs likely to have the greatest similarly to human response based on prior studies [80]. The anti-filovirus efficacy of remdesivir has been assessed in multiple NHP nonclinical studies, including an aerosol EBOV challenge study. Treatment of EBOV-infected NHPs with remdesivir results in a substantial improvement in clinical signs and symptoms and 80% to 100% survival regardless of the route of exposure [24]. Challenge viruses used in these studies included EBOV-Kikwit and MARV-Angola. Similar survival results were obtained with MARV-infected NHPs treated with remdesivir at the same dosing regimen as that used for EBOV NHP studies [36].

### j) The advantages of the Animal Rule for Marburg product development

Advantages of the Animal Rule include optimal modeling of target exposure including dose, route (aerosol), and timing of interventions over the course of one or more regulated GLP studies. Naïve, lab-reared animals lacking comorbidities also help to reduce confounding variables that complicate study interpretation. Study of AE is generally not possible during natural outbreaks. Less virulent filovirus outbreaks tend to be short-lived and relatively easily to contain when local healthcare teams are trained and equipped to respond. A prepositioned or rapidly deployable clinical team may be needed in the outbreak area to be able to conduct regulated trials serendipitously in most cases. Disadvantages of Animal Rule studies include the current shortage and increased cost of NHPs for research use due to export limitations in China and competing primate studies for COVID-19, relevance to naturally occurring disease where patients may often present later in the course of illness, rendering countermeasures requiring immediate application ineffective. Treatment windows may vary between patients and NHPs, and the large number of animals required in some cases may increase costs to the level of clinical trials. Viral kinetic modeling may address some of these limitations. Animal Rule studies also represent an opportunity to study disease processes in much greater depth with the use of routine necropsy in contrast to rare autopsies performed in VHF patients due to infection control considerations. They may help to inform the appropriate timing and/or trigger for intervention with the complementary combination therapies such as immunomodulators.

The advantages of developing a product with an existing commercial indication include a less expensive developmental pathway, a large safety database, existing GMP drug manufacturing, and supply chain for the existing commercial indication, which results in drug availability. Products developed solely for a biowarfare indication are developed for a small population and are thus likely to be expensive, may be in limited supply, and may require the USG to assume lead responsibility for bench-to-bedside product development in some cases. The massive increase in human safety and efficacy data and clinical experience with remdesivir coupled with greater manufacturing capacity will positively impact further development of remdesivir for filovirus infection. This will ensure broad availability of remdesivir for supplemental indications such as treatment MARV infection and may expedite the pathway to licensure.

## Discussion

Development of MCMs for biowarfare and rare disease indications are more expensive, time consuming, and riskier than starting development with a product already approved for a commercial indication. Developing an MCM without a commercial indication is a more difficult path than repurposing a drug that already has or will soon have an approved indication. With static or declining development budgets for biowarfare or rare disease indications in DOD, repurposing drugs with a commercial indication is an attractive method of delivering safe, approved MCMs for the warfighter. To fortify the emerging arsenal of therapeutic agents that might be employed in a deliberate bioterrorism attack, a broader range of viral threats could be evaluated and developed in NHPs under the Animal Rule. High-priority viral pathogens currently in need of safe and efficacious therapeutics include Marburg, Sudan-strain Ebola, Crimean-Congo, Lassa, Hanta, and other hemorrhagic fever viruses.

Animal models should be standardized where possible for comparability, allowing meaningful direct comparison of experimental outcomes. Investigators should drive for improved translational relevance by refining and benchmarking future studies with respect to previously published work. While limited studies—carefully planned and prepositioned—may be possible in outbreak settings, use of the animal rule is far more likely to be required given the epidemiology and relative rarity of sustained outbreaks of these diseases. These studies should also be planned with a view to establishing comparability and expanding on known clinical data to date.

Given the outcome of the PALM study, the clinical benefit of remdesivir relative to other therapeutics (mAB114, Regeneron EB3) was not sufficient to warrant continued development for treatment of EVD as monotherapy. The use of remdesivir in combination with mAb-based treatments has not yet been assessed in a clinical study, and combination therapy may well offer a clinical benefit particularly for patients who are diagnosed late in the course of their disease. Given poor efficacy in the late stage treatment cohort with mAB114 and REGN-EB3, there is clearly room for improvement to provide the best possible benefit for these patients.

Due to their high specificity, these recently developed monoclonal ZEBOV therapeutics are capable of being used to treat other Ebola species to include Sudan, Tai Forrest, and Bundibugyo. Of the remaining filoviruses without a clinical treatment, MARV is a logical choice for continued development given the severity of disease encountered in nature. MARV infections in nature have been shown to be highly lethal with case fatality rates up to 90%, and there are no MCMs available for treatment [2]. Monotherapy of a given antiviral drug alone may not be sufficient. Combination therapy consisting of 2 DAA drugs featuring complimentary mechanisms of action or different modalities (for example, small molecule and mAb) may be effective at extending the therapeutic window and/or treating advanced and severe disease.

Combination therapy between these agents (and remdesivir) had already been demonstrated in NHPs. A recent study in primates assessed the efficacy of a combination of remdesivir and a candidate Marburg mAb, and the combination of the two was more efficacious in treating late stage disease than either drug alone [37]. The benefits of such therapy should be explored for Ebola patients in the future. Clinical trials of mAbs, in combination with antiviral small molecules as adjunctive therapy, could be considered both for therapy of infected individuals and postexposure prophylaxis (PEP) in high-risk healthcare workers and other close contacts with infection exposure risk. As naturally occurring outbreaks of MARV are infrequent and difficult to predict, the product development pathway needed for a MARV treatment to obtain approval by the FDA will likely be through the Animal Rule.

The FDA created the Animal Rule as a pathway to develop medical countermeasures for indications that cannot ethically or reasonably be studied in humans. The intent of the Animal Rule is to provide a means to develop drugs and biological products to reduce or prevent life-threatening conditions caused by exposure to lethal diseases. Product efficacy under the Animal Rule is established based on adequate and well-controlled studies in animal models of the human disease, and safety is evaluated under the preexisting requirements for drugs and biological products [17]. The FDA will rely on evidence from animal studies when (1) there is a reasonably well-understood pathophysiological mechanism of the toxicity of the substance and its prevention or substantial reduction by the product; (2) effect demonstrated in more than one animal species with responses predictive for humans, unless there is an exceptionally well-characterized animal model; (3) the animal study endpoint is relevant to human disease; and (4) an effective human dose can be calculated based on available pharmacokinetic (PK) data.

Required studies under the Animal Rule include the following: (1) a Natural History study demonstrating pathology closely mimicking human disease; (2) PK studies to estimate the human-equivalent dose (HED); (3) a dose ranging study estimating the efficacious HED in animals; (4) at least 2 pivotal GLP animal studies at the estimated HED; and (5) often one or more postmarketing studies. To date, only 2 antiviral agents have been developed and approved under the Animal Rule—TPOXX and TEMBEXA—both for the treatment of smallpox. Both of these products used multiple animal species in well-developed models of poxvirus disease to demonstrate efficacy [81,82].

A number of outstanding questions remain moving forward in the development of anti-filoviral countermeasures. The recent development efforts related to remdesivir are illustrative. Timing is key, and it appears that earlier remdesivir administration leads to better outcomes. Patients enrolled in the PALM trial were treated an average of 6 days after the onset of symptoms, compared with 4 days after known experimental infection in the NHP models [22].

Finally, combination therapies should be considered in patients with moderate–severe Ebola and COVID-19. It is clear that remdesivir as monotherapy provided modest improvements. However, in patients with either disease in the advanced state, viral replication rapidly transitions to immune dysregulation. In that regard, late antiviral therapy in moderate–severe patients is likely to have less impact. Combination therapies with other therapeutic classes should also be considered to include both monoclonal therapies primarily targeting viral replication and immunomodulating agents. In the latter case, the recent ACTT-2 trial comparing remdesivir with and without baricitinib, a JAK1/2 immunomodulator, have shown modest additional reductions in mortality in hospitalized patients with severe COVID-19 not yet on mechanical ventilation [57]. However, this effect was less pronounced earlier in the course of illness when patients only required supplemental oxygen. The differences in outcomes based on timing of the interventions should also be explored in series and in parallel for severe disease.

Given a significant mortality reduction with mAbs in the PALM trial against Ebola, a combination trial with remdesivir should be considered. The advantages may include further mortality reduction, but perhaps more importantly, reduction in sequelae often observed in survivors. Viral persistence in immunologically privileged sites is thought to account for these phenomena. These can include persistent neurologic symptoms of headache, cognitive impairment, and fatigue, as well as persistence in semen with possible ongoing sexual transmission. The PREVAIL IV trial (NCT02818582) conducted in Guinea and Liberia was the first placebo-controlled study to evaluate clearance from immune-privileged sites with an antiviral. This relatively small study demonstrated reductions in the carrier state among male Ebola survivors [43]. Future studies should evaluate Ebola survivors for treatment of post-Ebola sequelae to include headache, fatigue, myalgia and arthralgia, memory loss and uveitis, and possible eradication of persistent virus in the central nervous system (CNS), eye, and elsewhere [83]. Moving forward, remdesivir therapeutic studies should evaluate earlier use against a broader spectrum of filoviruses, to include PEP in suspected contacts, such as healthcare workers. This approach is likely to improve survival rates for filovirus victims and improve outcomes in survivors.

## Key Learning Points

- The antiviral remdesivir is illustrative of the challenges and potential to develop broad-spectrum biowarfare countermeasures.
- Remdesivir was originally developed as an antiviral countermeasure against filovirus infection (Ebola). Remdesivir compared to monoclonal antibodies met the DOD’s requirement for efficacy but did not outperform 2 monoclonal antibody therapeutics in a recent head-to-head trial during a ZEBOV outbreak in the DRC.
- The US military has supported animal studies demonstrating the efficacy of remdesivir to treat MARV (a distinct filovirus species) infection and aerosolized EBOV infection.
- Remdesivir has since been found to have potential application against a host of other viral threats, most notably COVID-19, where it was recently licensed after demonstrated reductions in mortality and duration of hospitalization.
- While both the Ebola epidemic and COVID-19 pandemic allowed clinical testing of remdesivir, this is expected to be the exception, rather than the rule, for obtaining new product regulatory approval. Biodefense countermeasures will need to continue to rely on the FDA’s Animal Rule for future development. The Animal Rule is often the only path forward for FDA approval of medical countermeasures for biothreat agents where clinical trials cannot be conducted. Supportive clinical data should be sought where possible, considering ex vivo methods. Examples include in vitro antimicrobial inhibitory activity of human-dosed plasma or passive transfer of vaccine antibodies.

## Top Five Papers

1. U.S. Food and Drug Administration. FDA label for remdesivir. 2020. Available from: https://www.accessdata.fda.gov/drugsatfda_docs/label/2020/214787Orig1s000lbl.pdf
2. Mulangu S, Dodd L, Davey R Jr, Tshiani Mbaya O, Proschan M., Mukadi D, and the PALM Consortium Study Team. A Randomized, Controlled Trial of Ebola Virus Disease Therapeutics. N Engl J Med. 2019 Dec 12;381(24):2293–2303. doi: 10.1056/NEJMoa1910993. Epub 2019 Nov 27. PMID: 31774950.
3. Iversen P, Kane C, Zeng X, Panchal R, Warren T, Radoshitzky S, et al. Recent successes in therapeutics for Ebola virus disease: no time for complacency. Lancet Infect Dis. 2020 Sep;20(9):e231–e237. doi: 10.1016/S1473-3099(20)30282-6. Epub 2020 Jun 18. PMID: 32563280; PMCID: PMC7302789.
4. Madelain V, Baize S, Jacquot F, Reynard S, Fizet A, Barron S, et al. Ebola viral dynamics in nonhuman primates provides insights into virus immuno-pathogenesis and antiviral strategies. Nat Commun. 2018 Oct 1;9(1):4013. doi: 10.1038/s41467-018-06215-z PubMed PMID: 30275474; PubMed Central PMCID: PMC6167368.
5. Warren T, Jordan R, Lo M, Ray A, Mackman R, Soloveva V, et al. Therapeutic efficacy of the small molecule GS-5734 against Ebola virus in rhesus monkeys. Nature. 2016 Mar 17;531(7594):381–5. doi: 10.1038/nature17180. Epub 2016 Mar 2. Erratum in: ACS Chem Biol. 2016 May 20;11(5):1463. PMID: 26934220; PMCID: PMC5551389.
